# Patient adherence to antihypertensive medications in upper Egypt: a cross-sectional study

**DOI:** 10.1186/s43044-020-00066-0

**Published:** 2020-05-25

**Authors:** Ahmed Hussein, Mohammad Shafiq Awad, Hossam Eldin M. Mahmoud

**Affiliations:** 1 Department of Internal Medicine, Faculty of Medicine, Sohag University, Nasser City, Sohag 82524 Egypt; 2 Department of Cardiology, Faculty of Medicine, Beni Suef University, Beni Suef, Egypt; 3 Department of Internal Medicine, Faculty of Medicine, South Valley University, Qena, Egypt

**Keywords:** Hypertension, Antihypertensive medication, Adherence, Predictor factors

## Abstract

**Background:**

Control of hypertension is a very difficult issue. Non-adherence to medications is a well-recognized factor contributing to uncontrolled hypertension. It is required to detect factors that affect adherence of patients to antihypertensive medications at different societies and good planning with the collaboration of governments, universities, media, pharmaceutical companies, and civil society to create intervention programs ensuring good adherence to medications. In our study, we aimed to determine different factors affecting adherence to antihypertensive medications in Upper Egypt societies.

**Results:**

From September 2015 to September 2019, we conducted a large cross-sectional multi-center study among 2420 hypertensive patients attending the out-patient cardiac clinics at three different university hospitals. Data was collected through a personal interview with the patients using a questionnaire to cover a variety of items.

In the total of 2420 patients, we found that 1116 (46.12%) patients were adherent to medications and 1304 (53.88%) were non-adherent. From the final regression analysis of the results, we found that age > 65 years, illiterate patients, low income, associated comorbidities, using three or more antihypertensive pills, and living in rural areas were statistically significant socio-demographic factors associated with non-adherence to treatment. Also, missing doses of medication and lack of complying with dietary regimen were statistically significant behavioral causes associated with non-adherence.

**Conclusion:**

Many factors are predictors of good adherence to antihypertensive drugs, including young age, urban residence, a smaller number of pills, absence of comorbid conditions, high income, and high education level. Also missed doses of drugs and absence of complies with dietary regimen were the significant causes of non-adherence. Health institutions and governmental efforts should be directed toward improving adherence by creating effective intervention programs targeting these factors. Therefore, it might be concluded that patients who are more health ware are more adherent to medications than non-health aware patients.

## Background

Hypertension is one of the major preventable risk factors for increasing cardiovascular disease and stroke morbidity and mortality [1]. Treatment of hypertension needs both antihypertensive drugs and lifestyle modifications. Studies have detected that a significant number of hypertensive patients do not achieve the target levels for control of hypertension [2].

Non-adherence to antihypertensive medication is a major cause of uncontrolled hypertension [3]. Medication adherence is defined as to which extent the patients take their medication as prescribed by their health care providers [4]. Decreased adherence to medication is associated with increased morbidity and mortality in many chronic diseases [5–7]; therefore, it is important to assess the adherence to medication when interpreting clinical outcomes of chronic diseases. To improve the control rate of hypertension, it is important to increase patient adherence to antihypertensive medications.

Many studies evaluating factors affecting the adherence to medication were reviewed by Krousel-Wood et al. who concluded that medication adherence may be affected by many factors as sex, age, race, income level, education level, side effects of medication, patient knowledge, awareness, belief, attitudes, depression, and national health care system [8].

Detecting factors affecting adherence to medication in a certain population and developing intervention programs to increase antihypertensive medication adherence based on these factors are very important to increase the control rate of hypertension. Most of the existing studies for the assessment of adherence to antihypertensive medication were carried out on a small population or in small clinical settings, that is not enough to define the adherence rate of a larger population, such as national population, this is because measuring medication adherence is by using pharmacy refill records, and other measures such as electronic compliance monitoring, measuring of serum drug levels, physiologic drug effects, patient self-report, and clinician assessment can be applied only in clinical settings [9, 10].

This study was conducted to determine factors affecting antihypertensive medication adherence in Upper Egypt.

## Methods

### Aim of the study

This study was conducted to determine factors affecting antihypertensive medication adherence in Upper Egypt.

### Design of the study

From September 2015 to September 2019, we conducted a large cross-sectional multi-center study among 2420 hypertensive patients attending the out-patient cardiac clinics at three different university hospitals (Sohag, South Valley, and Beni Suef University Hospitals). All subjects provided a written informed consent to participate in the study. The study protocol was approved by the ethical committee at the universities where the study was conducted.

Data was collected through a personal interview with the patients using a modified non-adherence to treatment questionnaire [11] to cover a variety of items including socio-demographic factors (age, sex, marital status, occupation, education level, income, residence, number of pills, and associated comorbidities such as diabetes, dyslipidemia, and cardiovascular diseases) as predictors of adherence with medication regimen and behavioral factors (missing doses of medication, lack of motivation to be cured, not having enough time for exercise, lack of complying with dietary regimen, and lack of motivation to stop smoking) as causes of non-adherence with medication regimen. Adherence was calculated only for antihypertensive medications, and concomitant medications, such as for diabetes or dyslipidemia, were not considered.

Income was classified into three categories according to the individual income per year 1, low income (less than 8827 Egyptian pounds); 2, moderate income (from 8827 to 58900 Egyptian pounds); 3, high income (more than 58900 Egyptian pounds) [12, 13]. Level of education was classified into three categories 1, illiterate; 2, primary/secondary; 3, higher (university).

A pilot study was carried out on 300 persons chosen randomly (100 patients at each University Hospital) to estimate the time required for each interview and to identify difficulties that may arise, how to deal with and how to organize perfectly the field of work.

Medication regimen adherence was composed of 8 items, asking how often the patients forget to take their medicine: (1) How often do you forget to take your medicine? (2) How often do you stop taking your medicine because you feel better? (3) How often do you stop taking your medicine because you feel worse? (4) How often do you stop taking the medication because you believe that they are ineffective? (5) How often do you stop taking your medicine because you fear side effects? Or have caused side effect, dizziness/weakness. (6) How often do you stop taking medicine because you try to avoid addiction? (7) How often do you stop the medication because you are using traditional medicine (healer) or religious belief? (8) How often do you stop the medication because of cost of medication? The responses were measured on a 4-point Likert scale: (1) every day, (2) frequently, (3) rarely, and (4) never. Lifestyle modification adherence had 4 items: (1) How often do you smoke? (2) How often do you engage in physical exercise? (3) How often do you eat table salt? (4) How often do you eat meat with high animal fat? We excluded a question asking about alcohol consumption, as all the participants denied consuming alcohol at all, and this could result in a bias of the adherence rate, as all the participants will get 4 points for this question. In our community, even if the patient is consuming alcohol, he may deny that due to social, traditional, and religious factors. So, our questionnaire was composed of 12 items [10, 11]. Participants were asked to respond to the single question based on a 4-point Likert scale: how often desirable or undesirable behaviors relating to the control of hypertension. The responses were (1) every day, (2) frequently, (3) rarely, and (4) never. Some questions were set such that the response “every day” did not reflect the worst scenario of non-adherence, but it reflects the best scenario. To resolve this, these scores were reversed, for example, how often do you engage in physical exercise (4) every day, (3) frequently, (2) rarely, and (1) never

The 12 items measuring treatment adherence and lifestyle modification adherence were added up to get a sum index with a distribution ranging from 21 to 48 with mean 34 (SD = 5.31), and median split was 34, so the cutoff point of the adherence score for the participants was 34 (70%), which was dichotomized into two gatherings, i.e., 1—those who are non-adherent (≤ 34) and 2—treatment adherent (> 34).

The following formula was used to calculate the minimum size of the required sample for each of the three sites (universities) where the study was conducted:

$$ n={\left(\mathrm{z}\right)}^2\ p\left(1-\mathrm{p}\right)/{\mathrm{d}}^2 $$

where *n* indicates the sample size, *z* indicates the level of confidence according to the standard normal distribution (for a level of confidence of 95%, *z* = 1.96), *p* indicates the estimated proportion of the population that presents the characteristic (about 26%), *d* indicates the tolerated margin of error (for example, we want to know the real proportion within 5%).

Using the previous formula for the sample size calculation (*n*) = (1.96)^2^ × 0.26 (1−0.26)/(0.05)^2^ = 296. So, the minimum sample size is 296 participants.

### Participant characteristics

All hypertensive patients aged ≥ 18 year, taking antihypertensive treatment for at least 1 month ago and who agreed and consented to participate in the study were included.

### Analysis of data

The following formula was used to calculate the minimum sample size: *n* = (*z*)^2^ p (1−p)/d^2^ as prescribed in the “Methods” section.

The data was analyzed by the SPSS version 19 program for data entry and analysis; information was summarized using frequency tables and cross tabulations. The chi-squared test was used for categorical variables and *t* test for continuous variables. Univariate and multivariate binary logistic regression was done to detect socio-demographic and behavioral factors significantly associated with non-adherence. Odds ratio (OR) at 95% confidence interval (CI) and *p* values were computed. *P* value was considered significant at or below 0.05.

The outcome variable was treatment adherence, which is comprised of medication regimen and lifestyle modification adherence.

## Results

The study was conducted on 2420 hypertensive patients ≥ 18 years old attending cardiac outpatient clinic at Sohag, South Valley and Beni Suef University Hospitals.

**Fig. 1:**
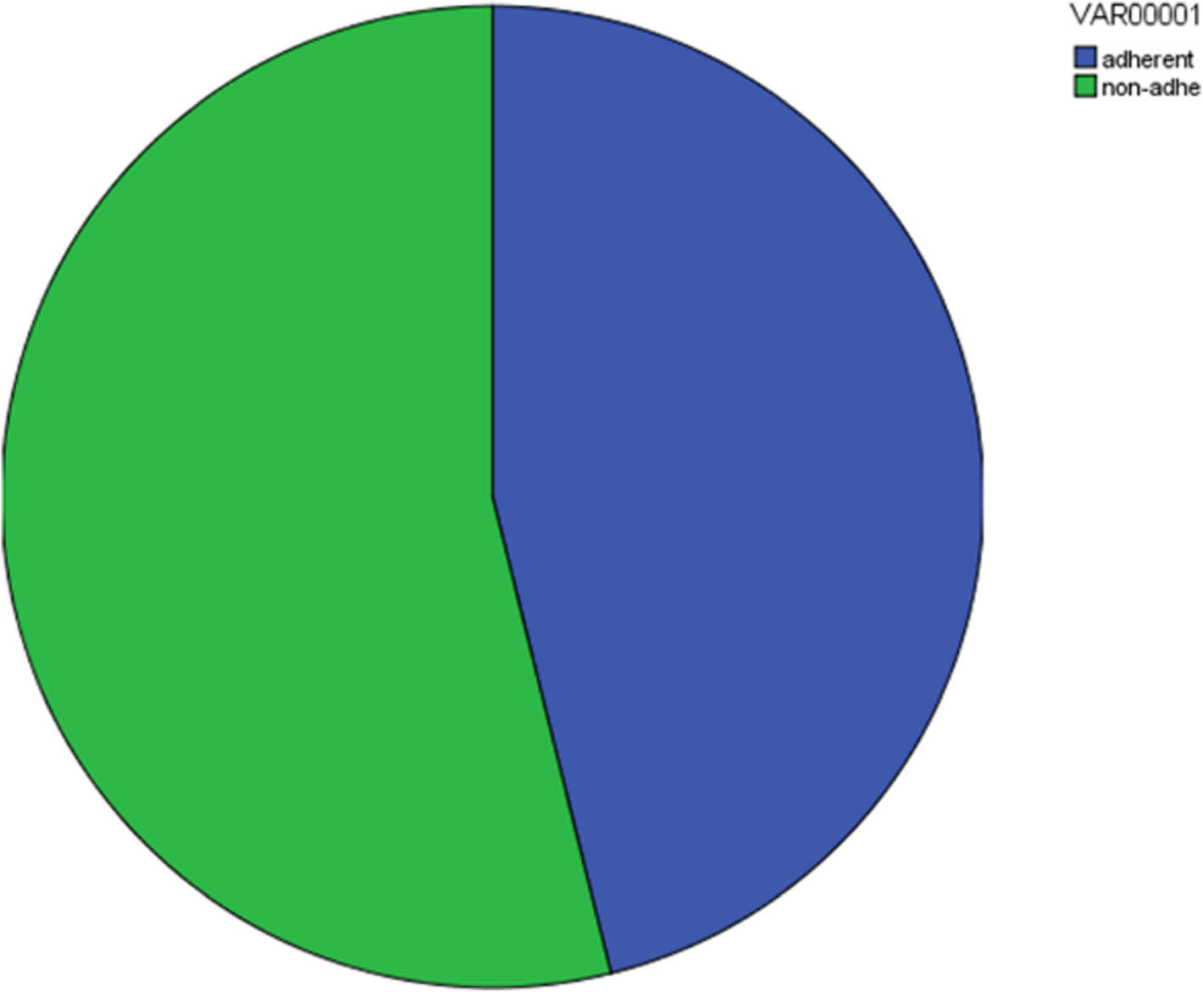
Adherence rate to medication: 46.12% were adherent and 53.88% were non-adherent

As regards adherence rate, 1116 (46.12%) patients were adherent to medications and 1304 (53.88%) were non-adherent (Fig. 1).

The socio-demographic predictors of adherence to medication from the final multivariate logistic regression analysis showed, younger age (< 40 years) was a significant predictor for adherence compared to patients (40-65 years) and patients > 65 years (OR, 7.25; 95% CI, 2.32-22.51; and OR, 2.56; 95% CI, 1.45-4.2 respectively; *p* value < 0.001). Patients with higher education level and patients with a primary/secondary level were more significantly adherent than illiterate (OR, 2.15; 95% CI, 1.02-4.45; and OR, 2.13; 95% CI, 1.21-3.9 respectively; *p* value 0.02). Also, higher income more significantly predicts the adherence than lower income (OR, 4.82; 95% CI, 2.01-9.03; and OR, 2.49; 1.21-4.91 respectively; *p* value < 0.001). Patients with other comorbid conditions were significantly less adherent to medications (OR, 2.21; 95% CI 1.14-4.34; *p* value 0.01). We found that patients using one or two antihypertensive pills were significantly more adherent than those using three or more pills (OR, 6.96; 95% CI, 2.71-17.71; and OR, 7.21; 95% CI, 2.63-19.71 respectively; *p* value < 0.001). Patients live in urban were significantly more adherent than those living in rural (OR, 2.97; 95% CI, 1.58-4.93, *p* value 0.04) (Tables 1, 2, 3, and 4).

**Table 1:** Distribution of baseline socio-demographic characteristics of participants

Characteristic	Treatment adherence		***p*** value
	Non-adherent	Adherent	
**Age**			< 0.001*
**< 40**	32 (14.3%)	192 (85.7%)	
**40-65**	436 (45.6%)	520 (54.4%)	
**> 65**	836 (67.4%)	404 (32.6%)	
**Gender**			< 0.001*
**Male**	664 (45.5%)	796 (54.5%)	
**Female**	640 (66.7%)	320 (33.3%)	
**Education level**			< 0.001*
**Illiterate**	560 (66.67%)	280 (33.33%)	
**Primary/secondary**	524 (50.58%)	512 (49.42%)	
**Higher**	220 (40.44%)	324 (59.56%)	
**Income**			< 0.001*
**Low**	768 (69.1%)	344 (30.9%)	
**Moderate**	400 (50.5%)	392 (49.5%)	
**High**	136 (26.4%)	380 (73.6%)	
**Comorbidities**			< 0.001*
**No**	208 (33.3%)	416 (66.7%)	
**Yes**	1096 (61%)	700 (39%)	
**No. Of antihypertensive pills**			
**One**	668 (44.7%)	828 (55.3%)	< 0.001*
**Two**	356 (61%)	228 (39%)	
**Three or more**	280 (82.4%)	60 (17.6%)	
**Marital status**			0.18
**Unmarried**	480 (53.3%)	420 (46.7%)	
**Married**	824 (54.2%)	696 (45.8%)	
**Residence**			< 0.001*
**Rural**	884 (59.25%)	608 (40.75%)	
**Urban**	420 (45.3%)	508 (54.7%)	
**Occupation**			0.42
**Unemployed**	440 (54.46%)	368 (45.54%)	
**Employed**	864 (53.6%)	748 (46.4%)	

Note: Significance of difference was measured by the chi-square test

*Significance at the 0.05 level or less

**Table 2:** Bivariate analysis of socio-demographic predictors of adherence to medication

Characteristic	OR	95% CI	***p*** value
**Age:**			< 0.001
**< 40**	9.02	3.64-22.47	< 0.001
**40-65**	1.96	1.31-2.98	< 0.001
**> 65**	1		
**Gender:**			
**Female**	1		
**Male**	2.04	1.36-3.05	< 0.001
**Education level**			< 0.001
**Illiterate**	1		
**Primary/secondary**	1.86	1.1-2.92	< 0.001
**Higher**	2.51	1.45-4.35	< 0.001
**Income**			< 0.001
**Low**	1	1	
**Moderate**	2.1	1.01-4.78	< 0.001
**High**	4.3	2.69-9.55	< 0.001
**Comorbidities**			
**No**	2.2	1.31-3.35	< 0.001
**Yes**	1		
**No. Of antihypertensive pills**			< 0.001
**One**	7.01	3.1-15.45	< 0.001
**Two**	8.75	3.68-20.57	< 0.001
**Three or more**	1		
**Marital status**			
**Unmarried**	1		
**Married**	1.15	0.76-1.71	0.59
**Residence**			
**Rural**	1		
**Urban**	2.7	1.53-3.9	0.02
**Occupation**			
**Unemployed**	1		
**Employed**	1.04	0.69-1.57	0.89

Note: Logistic regression

**Table 3:** Multivariate analysis of socio-demographic predictors of adherence to medication

Characteristic	OR	95% CI	***p*** value
**Age**			< 0.001
**<40**	8.1	2.4-25.85	< 0.001
**40-65**	2.63	1.3-4.64	< 0.001
**>65**	1		
**Gender**			
**Female**	1.82	1.01-3.35	0.14
**Male**	1		
**Education level**			0.01
**Illiterate**	1		
**Primary/secondary**	2.32	1.26-4.23	< 0.001
**Higher**	2.34	1.11-5.01	0.01
**Income**			< 0.001
**Low**	1		
**Moderate**	3.06	1.02-6.33	< 0.001
**High**	4.64	2.01-8.31	< 0.001
**Comorbidities**			
**No**	2.02	1.01-4.02	0.02
**Yes**	1		
**No. Of antihypertensive pills**			< 0.001
**One**	7.32	2.83-19.06	< 0.001
**Two**	7.64	2.72-21.66	< 0.001
**Three or more**	1		
**Residence**			
**Rural**	1		
**Urban**	2.5	1.75-4.86	0.03

**Table 4:** Final regression analysis of socio-demographic predictors of adherence to medication

Characteristic	OR	95% CI	***p*** value
**Age**			< 0.001
**<40**	7.25	2.32-22.51	< 0.001
**40-65**	2.56	1.45-4.2	< 0.001
**>65**	1		
**Education level**			0.02
**Illiterate**	1		
**Primary/secondary**	2.13	1.21-3.9	0.01
**Higher**	2.15	1.02-4.45	0.01
**Income**			< 0.001
**Low**	1		
**Moderate**	2.49	1.21-4.91	< 0.001
**High**	4.82	2.01-9.03	< 0.001
**Comorbidities**			
**No**	2.21	1.14-4.34	0.01
**Yes**	1		
**No. Of antihypertensive pills**			< 0.001
**One**	6.96	2.71-17.71	< 0.001
**Two**	7.21	2.63-19.71	< 0.001
**Three or more**	1		
**Residence**			
**Rural**	1		
**Urban**	2.97	1.58-4.93	0.04

The behavioral causes associated with non-adherence from the final regression analysis showed that, missing doses of medication and lack of comply with dietary regimen were the statistically significant behavioral causes associated with non-adherence (OR, 1.71; 95% CI, 1.27-2.32; and OR, 1.54; 95% CI, 1.23-1.96 respectively; *p* value < 0.001) (Tables 5, 6, and 7).

**Table 5:** Bivariate analysis of behavioral causes associated with non-adherence

Characteristic	OR	95% CI	***p*** value
**Missing doses of medication**	2	1.50-2.63	< 0.001
**Lack of motivation to be cured**	1.25	1.05-1.56	0.01
**Not having enough time for exercise**	1.07	0.9-1.37	0.58
**Lack of complying with dietary regimen**	1.69	1.3-2.11	< 0.001
**Lack of motivation to stop smoking**	0.81	0.63-1	0.06

**Table 6:** Multivariate analysis of behavioral causes associated with non-adherence

Characteristic	OR	95% CI	***p*** value
**Missing doses of medication**	1.74	1.31-2.41	< 0.001
**Lack of motivation to be cured**	0.82	0.6-1.11	0.21
**Lack of complying with dietary regimen**	1.61	1.32-2.09	< 0.001

**Table 7:** Final regression analysis of behavioral causes associated with non-adherence

Characteristic	OR	95% CI	***p*** value
**Missing doses of medication**	1.71	1.27-2.32	< 0.001
**Lack of complying with dietary regimen**	1.54	1.23-1.96	< 0.001

## Discussion

In the total of 2420 patients included in our study, we found that 1116 (46.12%) patients were adherent to medications and 1304 (53.88%) were non-adherent. We found that age > 65 years, illiterate patients, low income, associated comorbidities, using three or more antihypertensive pills, and living in rural areas were socio-demographic predictors of non-adherence. Missing doses of medication and lack of complying with dietary regimen were the behavioral causes significantly associated with non-adherence to treatment.

In our study, we found a lower adherence rate (46.12%) compared with that reported in previous studies conducted in other countries as China 2002 (51.7% adherence rate), the USA 2015 (67.6%), Korea 2018 (81.7%), and Pakistan 2007 (77%), and lower than the optimal rate of antihypertensive medication adherence estimated by the World Health Organization (WHO) in 2003 which ranges from 50 to 70%. But the rate of adherence in our study was comparable to the adherence rate found in a study conducted in Palestine in 2015 (adherence rate 45.8%) that reflects a poor health environment and culture in our societies [14–19].

In the elderly, we found that fewer patients are adherent to medications as compared to other elderly patients, which was similar to that shown in a cross-sectional study conducted in Turkey in 2012 [20]. While another study conducted in Saudi Arabia in 2015 showed that there was no relationship between age and medication noncompliance [21]. But a population-based study conducted in Bangladesh on 29,960 hypertensive patients in 2014 showed that compliance to medication increased as the age increased [22]. Other studies in different countries reported that older age was shown to be associated with better treatment adherence among hypertensive patients and this was explained by the perceived susceptibility and severity of the disease [16, 23–25]. Our result could be explained by the absence of good geriatric health care programs and geriatric clinics in Upper Egypt.

In our study, the level of education was a significant predictor of medication adherence, as the patients with higher education level showed a higher adherence rate. Similar results were reported by other studies conducted in different developing countries as Pakistan in 2007, Palestine in 2013, and the United Arab Emirates in 2015 [17, 26, 27]. Nunes et al. reported in their study that there was no association between education level and treatment compliance [28].

Higher income was significantly associated with good adherence to treatment in our study matched with the results of some studies as Vawter et al., Herttua et al., Khanam et al., and Abdulazeez et al. that reported a significant association between medication adherence and patients’ financial situation [22, 29–31]. This is explained by that; poor patients struggle to adhere to medications due to their cost.

The presence of comorbid conditions in hypertensive patients was associated with non-adherence to treatment as shown in our study that agrees with the results of a previous study conducted in the United Arab Emirates by Bader et al., and another study by Jose et al. [27, 32]. This is explained by that hypertensive patients without other comorbidities have a simple treatment regimen that makes adherence easier. In contrast, other studies conducted by Hyo Yoon Choi et al., Mallya SD et al., and Mazzaglia G et al. [16, 33, 34] showed a significant association between the presence of comorbid conditions in hypertensive patients and good adherence to antihypertensive medications and explained that patients with concomitant comorbid conditions, especially those related to cardiovascular risk factors, are more likely to be aware of being at a higher risk and, therefore, more likely to adhere to a therapeutic regimen.

We found that patients using three or more antihypertensive pills were significantly less adherent to medications than those using only one or two drugs, and this finding was in line with the results of the study conducted in Pakistan by Hashmi et al., and the study by Jose et al. [17, 32]. This could be explained by a smaller number of medications is easier to remember and less side effects lead to more adherence than multiple medications. On the other hand, some previous descriptive studies showed a positive association between adherence and the number of pills, as the study conducted by Hyo Yoon Choi et al. and Hashmi et al. [14, 25]. They explained that by patients who are using multiple pills tend to be more motivated to take medications due to the perception of the severity of the disease. A second explanation may be that patients who are using multiple drugs daily are less likely to forget them compared to those using only one drug.

Our study was in line with the previous two studies conducted in South Korea by Park JH. et al. and Hyo Yoon Choi et al. [16, 35], who reported that there is a higher medication adherence rate in patients who were treated in metropolitan rather than in urban clinics and this is explained by that medical services are focused around metropolitan areas. Also, in our study the adherence rate was significantly higher in patients living in urban than those living in rural areas. This is explained by the high gap in health and education services between urban and rural areas in our communities in favor of urban.

A previous study conducted in Saudi Arabia by Mahmoud M. showed a significant association between poor adherence and smoking [36]. This could be explained by those patients with good quality of life showed a good adherence to their medications, as they are aiming to have a better health, so they try to keep a good adherence to their medications [37]. In contrary, our study showed no association between smoking and non-adherence to medication.

We noticed that missing doses of medication were a significant cause of non-adherence. Larissa Grigoryan et al. [38] concluded that, regardless of the number of medications, non-adherence to antihypertensives is usually partial, with most patients engaging in drug holidays of less than 4 days.

In our study, patients with lack of complying with a dietary regimen showed non-adherence to antihypertensive medication. The observational study conducted in India in 2015 [39] reported that poor adherence to treatment was observed in patients with unrestricted dietary habits and a high salt intake that matched with our results. Another study with similar findings conducted by Natarajan N. et al. [25] noticed that patients who had a healthy diet with a low salt intake had a good adherence to medication. Also, Hyo Yoon Choi et al. [16] showed that patients with high salt intake had poor adherence to antihypertensive medication. These results suggest that a patient’s lifestyle modification enhances good adherence to medications.

*The limitation of our study*. We used an indirect method for evaluation of the factors that predict adherence through the patient’s interview using adherence questionnaire, which is less accurate than the direct methods measuring the drug concentration or its metabolite in body fluids as self-reporting of treatment adherence could introduce recall bias by either over reporting or under reporting depending on the patient’s behavior on the recent past.

*Strengths of our study*. The first, it included a large number of patients. The second, it was a multicenter study conducted at three different governates that represented different societies.

## Conclusion

In our study, the adherence rate to medication was 46.12%. Age > 65 years, illiterate patients, low income, associated comorbidities, using three or more antihypertensive pills, and living in rural areas were socio-demographic predictors of non-adherence to treatment. Also, missing doses of medication and lack of complying with dietary regimen were the statistically significant behavioral causes associated with non-adherence. Health institutions and governmental efforts should be directed toward improving adherence by creating effective intervention programs targeting these factors. Therefore, it might be concluded that patients who are more health aware are more adherent to medications than non-health aware patients.

## Data Availability

The datasets used and/or analyzed during the current study are available from the corresponding author on reasonable request.

## Abbreviations

SD: Standard deviation
OR: Odds ratio
CI: Confidence interval
NO.: Number
WHO: World Health Organization
